# Spatiotemporal Distribution Patterns of Plateau Foxes and Their Lagomorph Prey in Baqing County, Tibet

**DOI:** 10.1002/ece3.73361

**Published:** 2026-03-30

**Authors:** Yuanzhen Cui, Dandan Wang, Fuxing Huang, Zhiming Cao, Yuqin Liu, Awang Pingcuo, Da Qiong, Xiaolong Hu, Yongtao Xu

**Affiliations:** ^1^ Jiangxi Provincial Key Laboratory of Conservation Biology Jiangxi Agricultural University Nanchang China; ^2^ The Bureau of Natural Resources of Baqing County Baqing Tibet China; ^3^ College of Animal Science and Technology Jiangxi Agricultural University Nanchang China

**Keywords:** activity rhythms, camera‐traps, occupancy models, spatiotemporal co‐occurrence, sympatric coexistence

## Abstract

Predation by carnivores is a fundamental driver of species evolution, shaping interspecific spatiotemporal dynamics and prey behavior. To examine predator–prey‐driven spatiotemporal coexistence patterns between foxes and pikas on the Qinghai‐Tibetan Plateau (QTP), we conducted an infrared camera‐trapping survey in Baqing County, Tibet. From July 2023 to May 2024, we monitored two mesocarnivores (Tibetan fox and red fox) and their principal prey (plateau pika and Glover's pika) across cold and warm seasons. Both pika species exhibited higher relative abundance during the warm season than during the cold season. Kernel density estimation indicated that the Tibetan fox was predominantly diurnal, whereas the red fox was primarily nocturnal, resulting in low diel activity overlap between the two species (Δ < 0.5). The Tibetan fox showed high temporal overlap with both pika species during the warm season, whereas the red fox consistently exhibited lower overlap with its prey. Spatial niche overlap among the four species remained low (< 0.2) across both seasons. Conditional occupancy models further revealed that Tibetan fox occurrence was positively associated with plateau pika presence, whereas red fox occurrence declined in areas where both pika species were present. These patterns likely reflected interspecific differences in foraging strategies and reliance on anthropogenic food subsidies, which reduced interference competition and facilitated coexistence through differential prey use and spatiotemporal niche partitioning. Collectively, our findings advanced understanding of the mechanisms underlying sympatric coexistence on the QTP and provided important implications for biodiversity conservation and grassland ecosystem management.

## Graphical abstract

Our study suggests pronounced spatiotemporal niche differentiation as a mechanism facilitating two mesocarnivores' coexistence, and enhances our understanding of the mechanisms underlying sympatric coexistence on the Tibetan Plateau.

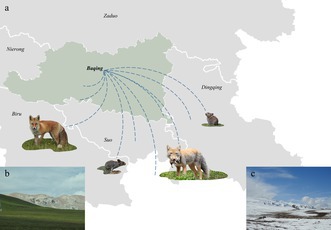

## 1 Introduction

Predators acquire energy and nutrients by hunting and consuming prey, thereby enabling the transfer of matter and energy across different trophic levels (Hairston Jr and Hairston Sr 1993). As key predators in food webs, carnivore guilds play a vital role in ecological communities by generating trophic cascades, facilitating energy and nutrient flow, and influencing food web stability (del Rio et al. 2001; Lesmeister et al. 2015). Prey availability can influence interactions among predators, thereby constraining species distributions. Within the same geographic region, coexisting species with similar body sizes and substantial dietary overlap typically experience the most intense competition, which may lead to both interference and exploitative competition, particularly pronounced among carnivore species (Havmøller, Parsons, et al. 2024; Havmøller, Wahyudi, et al. 2024). Resource competition drives species evolution, alters foraging strategies, and shapes the complex spatiotemporal patterns of species distributions (Venturino and Petrovskii 2013).

Predator–prey interactions are a major driving force behind species evolution and survival strategies, and their dynamics are shaped by the interplay of individual behavioral traits, habitat structure, resource availability, and the demographic state of the species involved (Schmitz 2017). To minimize negative interactions, carnivores often adjust their spatiotemporal behaviors to achieve risk avoidance and niche segregation (Monterroso et al. 2020). A study on the red fox ( *Vulpes vulpes* ), the sable ( *Martes zibellina* ), and the pine marten ( *Martes martes* ) found that although the three species showed high overlap in habitat selection and diet, they reduced the likelihood of direct encounters by shifting their activity peaks by 1–4 h (Petrov et al. 2016). Similarly, research on coyotes ( *Canis latrans* ) and gray foxes ( *Urocyon cinereoargenteus* ) in North America has demonstrated that temporal partitioning is a key mechanism promoting stable species associations; gray foxes tend to reduce activity during periods of intense coyote activity as a means of disturbance avoidance (Rodríguez‐Luna et al. 2024). In sympatric carnivore communities, spatial and dietary segregation also play critical roles. Although golden jackals ( *Canis aureus* ) and the red fox exhibited similar daily activity rhythms, red foxes alleviated competition by actively avoiding areas heavily used by jackals and by displaying greater dietary generalism (Torretta et al. 2021). In the case of coyotes and bobcats ( *Lynx rufus* ), which both preyed primarily on mountain beavers ( *Aplodontia rufa* ) and snowshoe hares ( *Lepus americanus* ), dietary similarity was extremely high (Pianka's overlap index = 0.97), yet the two species partitioned and utilized habitats along different elevational gradients (Witczuk et al. 2015). Moreover, human activities have increasingly become a dominant force shaping coexistence patterns among canids, driving shifts in wildlife activity rhythms, spatial use, and even restructuring their niche configurations (Lewis et al. 2021).

**FIGURE 1:**
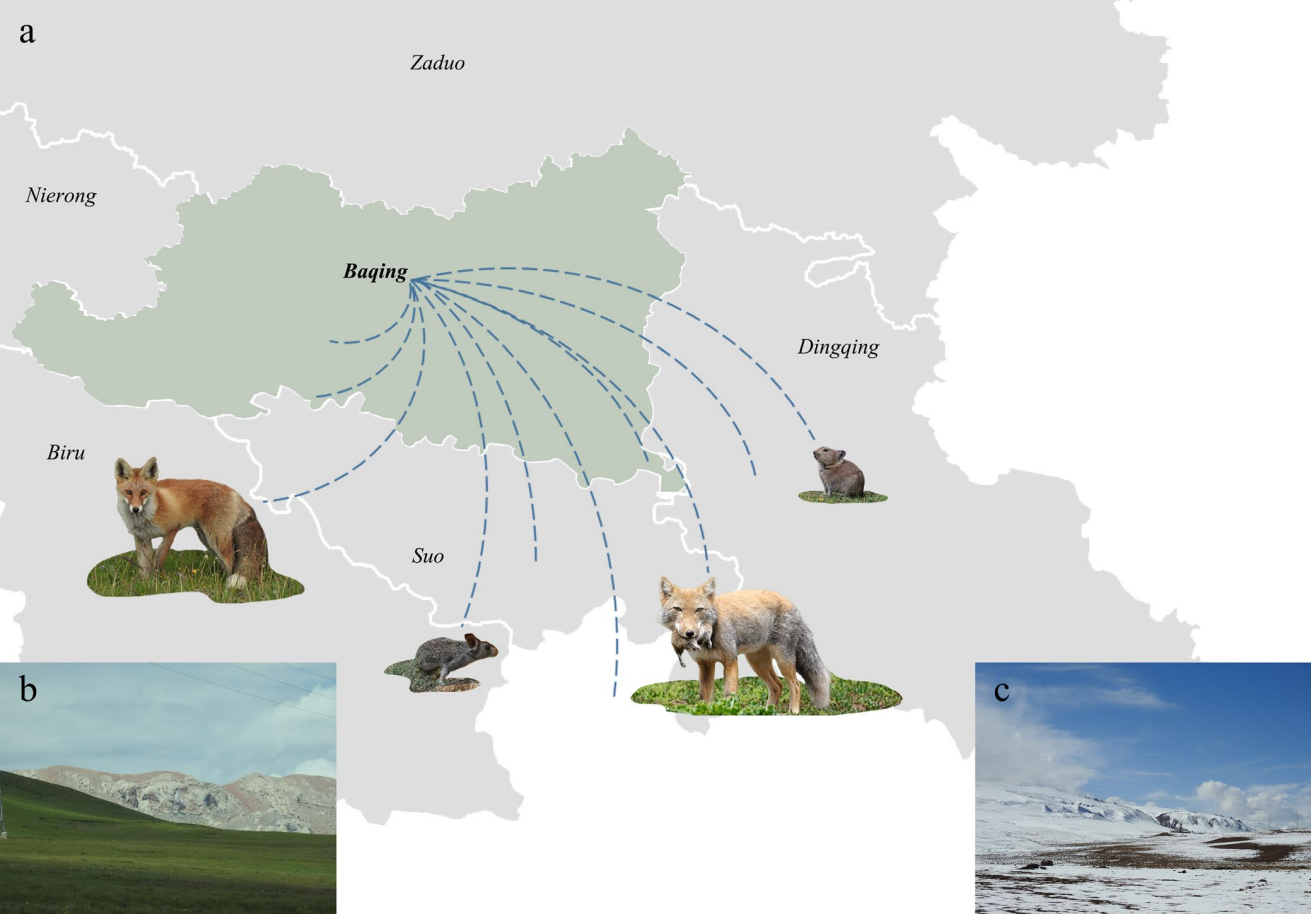
Study area of mesocarnivores and main prey species: (a) The boundary of Baqing County (b) The ecological photo of warm season (c) The habitat in cold season.

The Tibetan fox ( *Vulpes ferrilata* ) and red fox ( *V. vulpes* ) are classified as National Class II protected species in China and are listed as Least Concern (LC) on the IUCN Red List (Hoffmann and Sillero‐Zubiri 2021). The red fox is thought to have originated in the Middle East approximately 2.38 million years ago and subsequently expanded across Europe, Asia, and North America (Statham et al. 2014). In contrast, the Tibetan fox, an endemic species of the Qinghai‐Tibetan Plateau (hereafter, QTP), has adapted to high‐altitude environments earlier than the red fox and also serves as a key definitive host for *Echinococcus shiquicus* (Zhao et al. 2016; Chen et al. 2025). The divergence of the two species was estimated to have occurred 48,000–49,000 years ago, coinciding with the initial uplift phase of the Qingzang Movement, a significant geological event associated with the uplift phases of the QTP (Dai 2004). In the alpine grassland and desert ecosystems of the QTP, the Tibetan fox and red fox functioned as mesoconsumers, occupying the upper‐middle levels of the food web. They primarily fed on small lagomorphs, particularly plateau pikas ( *Ochotona curzoniae* ) (Liu et al. 2010), while also consuming birds, *Lepus oiostolus* , and other available food resources. Both species played key roles in controlling grassland rodent outbreaks (Figure 1), maintaining biodiversity, and facilitating energy flow within the ecosystem (Schaller 2000). However, historically, illegal poaching driven by the fur trade, together with habitat destruction, led to population declines of the Tibetan fox and red fox over recent decades (Clark et al. 2008). Thus, understanding the complex relationships between the two species is imperative for effective ecosystem management and species conservation (Rød‐Eriksen et al. 2023).

In recent years, camera trapping has been increasingly used to study wildlife ecology and inform conservation efforts (O'Connell et al. 2011). This method has proven effective for estimating population densities of cryptic and low‐density species (e.g., *Dasyurus maculatus gracilis*), quantifying mammalian activity rhythms and assessing species diversity across different habitats (Rowland et al. 2023; Mu et al. 2019; Stein et al. 2008). A class of occupancy models has proven useful for modeling factors influencing species occupancy probabilities under imperfect detection (Nichols et al. 2007). In Texas, multispecies occupancy models were applied to examine interspecific interactions and the effects of patch‐and landscape‐level metrics on the occurrence of ocelot ( *Leopardus pardalis* ), bobcat ( *Lynx rufus* ), and coyote, revealing that seasonal coexistence patterns were associated with increasing distance from high‐speed roadways (Lombardi et al. 2020). Using occupancy modeling incorporating spatial autocorrelation, Wang et al. (2019) assessed habitat use for ocelots across the Brazilian Amazon, and found that occupancy probabilities were often influenced by the presence or absence of interacting species.

Previous studies on the Tibetan fox and red fox have mainly focused on den‐site selection (Wang et al. 2008), parasites (Jiang et al. 2012; Shang et al. 2021), genome assembly (Zhao et al. 2016; Lyu et al. 2022), and diet (Soe et al. 2017). However, it remains unclear whether the spatial distribution and habitat selection of the Tibetan fox and red fox are influenced by the presence of prey or competing species, as few studies have specifically examined the predation relationships and coexistence mechanisms between these foxes and small lagomorphs in plateau ecosystems. The plateau pika and Glover's pika ( *Ochotona gloveri* ), small burrowing lagomorphs endemic to the QTP, are abundant and constitute important food resources for the Tibetan fox and red fox (Wang et al. 2025; Zheng et al. 2023). This study aims to explore the complex spatiotemporal distribution patterns of the Tibetan fox, red fox, and their pika prey, and assess the potential effects of environmental factors on the occurrence and distribution of these species in Baqing County on the QTP. Specifically, we seek to elucidate their temporal activity patterns, spatial distribution characteristics, and the key environmental drivers shaping interspecific co‐occurrence, thereby providing a scientific basis for maintaining food‐chain functional balance and promoting predator–prey coexistence within the ecosystem.

## 2 Materials and Methods

### 2.1 Study Area

Baqing County (93°55′49″ E, 32°35′41″ N) is located in eastern Nagqu Prefecture, Tibet Autonomous Region, within the upper Nujiang River basin and the southern Qiangtang Lake basin on the northern QTP. It is part of the core ecological zone of the Sanjiangyuan region. The area is characterized by a cold, high‐altitude plateau monsoonal climate, with approximately 2402 h of annual sunshine, a mean annual temperature of 1.7°C, and mean annual precipitation ranging from 500 to 600 mm (Fu et al. 2019). The study area covers 9811 km^2^, with higher elevations in the north and lower elevations in the south, an average elevation exceeding 4500 m, diverse landforms and ecosystems, and vegetation exhibiting distinct altitudinal zonation, such as plateau shrublands, wetlands, and grasslands (Peng et al. 2025). These unique geographic and climatic conditions support a diverse wildlife community.

### 2.2 Infrared Camera Deployment Method

**FIGURE 2:**
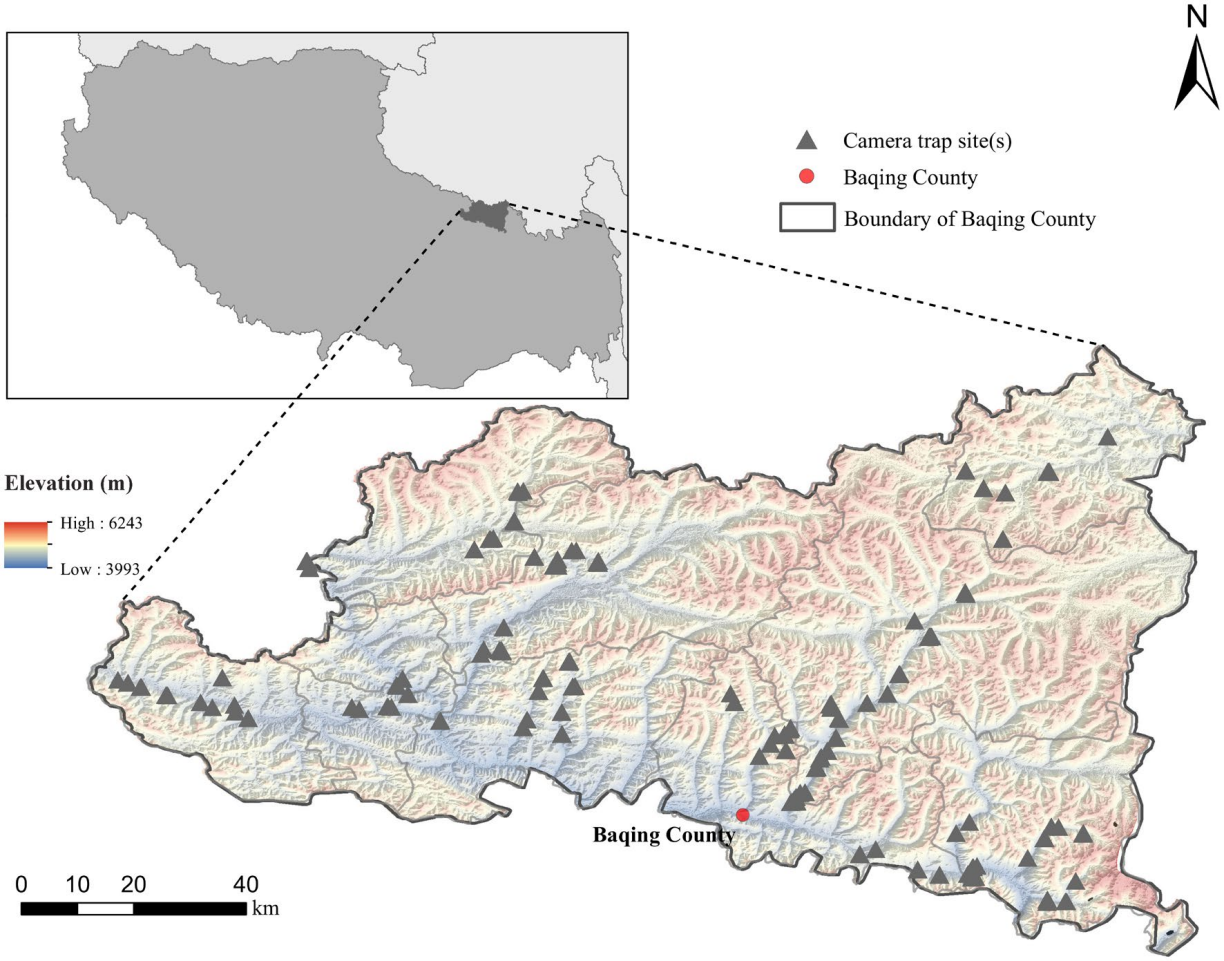
Spatial distribution of camera trap sites in Baqing County, Tangbei region of Sanjiangyuan.

Between July 2023 and May 2024, a total of 223 infrared camera traps were deployed across all townships in Baqing County (Figure 2). To ensure systematic and comprehensive spatial coverage of the study area, we implemented a 5 × 5 km grid‐based sampling design using ArcGIS 10.8. Camera trap locations were selected based on: (1) covering different habitat types, including alpine meadow, alpine shrubland, riverine wetlands, marshes, and alpine mountainous areas; (2) representing a range of altitudinal gradients and topographic variation; (3) ensuring field accessibility and feasibility of camera installation; and (4) evidence of frequent wildlife activity, such as trails, feces, and footprints (Xu et al. 2022).

Owing to the open terrain and the lack of natural mounting structures, most camera traps were mounted on stones or wooden stakes (30 × 30 × 80 cm), while a smaller proportion were installed on shrubs. All camera lenses were generally aligned parallel to the slope to minimize blank captures caused by direct sunlight, and any obstructing vegetation or objects in front of the cameras were removed. Each image captured by the cameras was stamped with the date, time, and ambient temperature, and the geographic coordinates of each camera location were recorded using GPS. To minimize human disturbance to camera detection rates, batteries and memory cards were replaced at 3‐month intervals, and camera placements at poorly performing sites were adjusted to improve image quality and detection efficiency.

Infrared‐triggered cameras (Yi'an Guardian L730 Series, China) were placed at a height of 30–60 cm above the ground and configured as follows: (1) the monitoring mode was set to “3 photos + 1 video” (20s duration), with a 0 s interval between consecutive videos; (2) still images were recorded at a resolution of 12 megapixels, and were recorded at 1920 × 1080 pixels; (3) the passive infrared sensor (PIR) sensitivity was set to “high”; (4) camera‐trap data were categorized into warm (May–September) and cold (October–April) seasons for each year.

### 2.3 Relative Abundance Analysis

The relative abundance index (RAI), derived from the number of independent camera‐trap detections per species, was used as an indicator of relative population abundance (Palmer et al. 2018). One effective camera‐trap day was defined as 24 h of continuous camera operation, and RAI values were standardized by the total number of effective camera trap days accumulated over the 100‐day survey period. For each camera and species, consecutive valid photos of the same species taken within a 30‐min interval were considered a single independent detection to minimize recounting the same individual multiple times (O'Brien et al. 2003). The formula is as follows:

(1) $$\mathrm{RAI}=\frac{A_{i}}{N}\times 100$$

In the formula, *A*_*i*_ represents the number of independent detection events of the target species *i*, and *N* denotes the total number of effective camera‐trap days (O'Brien 2011).

### 2.4 Activity Rhythm Analysis

To assess nocturnal activity patterns, we calculated the nocturnality index following. In the study area, sunrise occurs between 07:00 and 09:00, and sunset occurs between 19:00 and 21:00 throughout the year. Solar time conversion was applied to calculate the nocturnality index. Species were categorized as diurnal (*β* < 0.54), nocturnal (*β* > 0.54), or cathemeral (*β* = 0.54), the latter indicating an absence of a pronounced diel activity pattern (Gómez et al. 2005; van Schaik and Griffiths 1996). The formula is as follows:

(2) $$\beta =\frac{D}{N}\times 100\%$$

In the formula, *β* represents the nocturnality index; we denote the nocturnality indices for the warm and cold seasons as *β*_*ws*_ and *β*_*cs*_, respectively. *D* is the number of independent events of the target species recorded during the nighttime period (21:00–07:00); *N* is the total number of independent events of the target species recorded across all time periods (Pearson 1947).

Kernel density estimation was used to characterize the diel activity patterns of each species (Linkie and Ridout 2011). Using a continuous daily cycle as the temporal framework, the timestamps (hh:mm:ss) of independent valid photos were first converted into decimal values ranging from 0 to 1 and then transformed into radians. The Hermans‐Rasson uniformity test was performed using the CircMLE package to assess whether the activity patterns of each species were randomly distributed throughout the diel cycle (Fitak and Johnsen 2017; Landler et al. 2019). For small sample sizes (< 50 records), Δ₁ was used, whereas Δ₄ was applied for larger sample sizes (≥ 50 records). Sample sizes for Tibetan fox (hereafter, TF), red fox (hereafter, RF), plateau pika (hereafter, PP), and Glover's pika (hereafter, GP) were all greater than 50 individuals, allowing the use of the Δ4 estimator in analyzing activity patterns. Diel activity rhythms were visualized using the densityPlot() function, and temporal overlap between species pairs was visualized using the overlapPlot() function from the *overlap* package in R (version 4.4.2). The coefficient of overlap (Δ) between each species pair was quantified on a scale from 0 (no temporal overlap) to 1 (complete temporal overlap). The 95% confidence intervals of Δ were obtained through 1000 bootstrap samples using the compareCkern() function from the activity package. Values of Δ ≥ 0.8 were considered high overlap, whereas 0.5 ≤ Δ < 0.8 indicated moderate overlap (Mulekar and Mishra 2000; Schmid and Schmidt 2006; Ridout and Linkie 2009).

### 2.5 Spatiotemporal Niche Analysis

Given the variation in sampling effort across sites, niche breadth and overlap were calculated using Levin's index (Levins 1968) and Pianka's index (Pianka 1973), and all analyses were performed in R using the spaa package (Gotelli 2000). The corresponding formulas are presented below as Equations (3) and (4).

(3) $$B=1/\sum_{i=1}^{n}p_{i}^{2}$$

(4) $$Q_{\mathrm{jk}}=\frac{\sum_{i=1}^{r}P_{\mathrm{ij}}P_{\mathrm{kj}}}{\sqrt{\sum_{i=1}^{r}P_{\mathrm{ij}}^{2}\sum_{i=1}^{r}P_{\mathrm{kj}}^{2}}}$$

In Equation (3), *B* represents the niche breadth of the target species according to Levin's index, where *P*_*i*_ is the proportion of the species' RAI at site i relative to the total RAI across all camera‐trap sites, and *n* is the total number of sites. In Equation (4), *Q*_*jk*_ is Pianka's niche overlap index between species *j* and *k* according to Pianka's index. Here, *P*_*ij*_ represents the RAI of species *i* at site *j*, *P*_*kj*_ represents the RAI of species *k* at site *j*, and *r* denotes the total number of sites. All analyses were conducted using R version 4.4.1.

### 2.6 Occupancy Model Analysis

In this study, we conducted spatial autocorrelation analysis prior to the occupancy modeling and excluded 44 camera sites. Ultimately, 170 camera locations were retained, with a minimum inter‐camera distance of 300 m (Sarmento et al. 2009; Cove et al. 2013; Morin et al. 2022). We applied single‐season multi‐species occupancy models (MSOMs) to assess the spatial niche states of TF, RF, PP, and GP, which assume the latent occupancy state was a multivariate Bernoulli random variable (Dai et al. 2013) and allowed construction of numerically stable species co‐occurrence covariate models that did not require a priori assumptions of asymmetric interactions (i.e., one species being dominant over the other) (Rota et al. 2016). Previous studies on canids have demonstrated that both natural factors and human activities, such as elevation, roads, and human settlement, can substantially influence fox activity patterns and habitat use (Liu et al. 2007; Wang et al. 2007; Reshamwala et al. 2021). Accordingly, five variables were included as covariates in our analysis: distance to the nearest settlements, distance to the nearest road, as well as slope, aspect, and altitude at each camera‐trap location.

Model implementation followed three processes. Model 1 reflected the hypothesis that all four species occur independently and that marginal occupancy probabilities for each species were a function of temperature and a single covariate (Table S1). Model 2 built on Model 1 and additionally reflected the hypothesis that species exhibited constant pairwise dependence (Table S2). Model 3 reflected the hypothesis that the relationship between covariates and occupancy probabilities for each species varied in the presence and absence of each of the other species (Table S3). For model selection, we used the top‐ranked detection intensity model to determine the best set of spatial (occupancy) predictors (Table S4), and the candidate models were subsequently evaluated and compared using the Akaike Information Criterion (AIC).

Occupancy models were fitted using the *unmarked* package in R version 4.4.2 (Fiske and Chandler 2011). Detection and site‐level covariates were standardized and formatted for analysis using the *unmarkedFrameOccuMulti* function (see Rota et al. 2016 for model code). All models achieved adequate convergence by running for 2000 iterations following a burn‐in phase of 2000 iterations. Models producing non‐estimable parameters (NaN) or excessively large standard errors were excluded from further consideration. Two site‐level covariates (aspect and altitude) were removed from the analysis because their inclusion consistently led to non‐convergence and invalid parameter estimates. Consequently, only four covariates, slope, distance to the nearest settlement, distance to the nearest road, and yak abundance, were retained for subsequent analyses.

## 3 Results

### 3.1 Species Relative Abundance

Of the 223 deployed camera traps, nine were lost, and monitoring data were collected from the remaining 214 traps, totaling 17,062 camera trap days and yielding 12,848 independent events. In this study, we focused on the two fox species (TF, RF) and their important lagomorph prey (PP, GP).

During the warm season, the GP exhibited the highest number of independent events and RAI (*n* = 819, RAI = 70.112), followed by the PP (*n* = 699, RAI = 43.585), RF (*n* = 563, RAI = 12.035), and TF (*n* = 108, RAI = 7.155). In the cold season, predator RAI values increased slightly, whereas prey abundance declined markedly, with GP (*n* = 130, RAI = 35.845) and PP (*n* = 546, RAI = 21.905). Meanwhile, the RF (*n* = 382, RAI = 13.790) and the TF (*n* = 71, RAI = 8.034) were recorded (Table 1).

**TABLE 1:** Relative abundance and detection metrics of Tibetan fox, red fox, plateau pika, and Glover's pika during the cold and warm seasons in Baqing County.

Order	Family	Species	Number of independent events	Number of camera sites	Relative abundance index	
					Warm season	Cold season
Carnivora	Canidae	Tibetan fox	179	53	7.155	8.034
		Red fox	945	170	12.035	13.790
Non‐carnivores	Ochotonidae	Plateau pika	949	29	43.585	21.905
		Glover's pika	1245	55	70.112	35.845

### 3.2 Seasonal Activity Patterns

During the warm and cold seasons, based on the nocturnality index (*β*), the TF (*β*_*ws*_ = 0.269, *β*_*cs*_ = 0.085), PP (*β*_*ws*_ = 0.103, *β*_*cs*_ = 0.185), and GP (*β*_*ws*_ = 0.204, *β*_*cs*_ = 0.385) were identified as diurnal species in both seasons, whereas the RF exhibited pronounced nocturnality (*β*_*ws*_ = 0.737, *β*_*cs*_ = 0.581). The Hermans‐Rasson test indicated that activity patterns of all species were significantly clustered rather than randomly distributed (*p <* 0.05). Seasonal differences in diel activity were evident across all four species. Temporal overlap between the TF and RF remained low, with overlap coefficients of 0.50 (95% CI: 0.49–0.51) in the warm season, and 0.46 (CI: 0.43–0.46) in the cold season. During the warm season, the TF exhibited a primary activity peak around 14:00 and a secondary peak near 21:00. In contrast, the RF exhibited nocturnal activity, with peak activity occurring around 22:00. In the cold season, the TF's activity peak shifted to approximately 15:00, about 1 h later than in the warm season. Meanwhile, the RF became more active near midday (around 12:00), resulting in a slight increase in temporal overlap.

**FIGURE 3:**
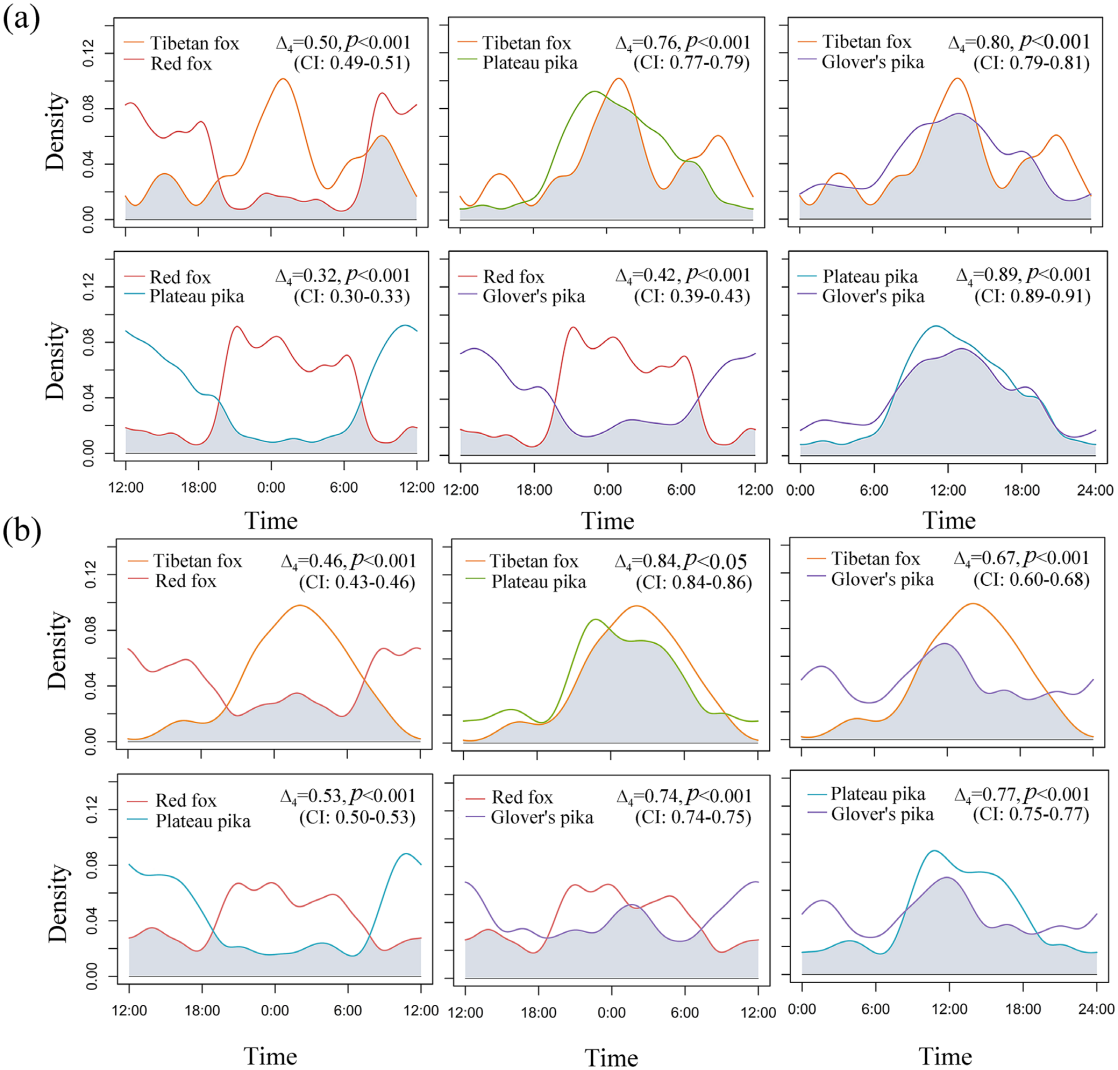
Daily activity overlap curves of Tibetan fox, red fox, plateau pika, and Glover's pika in Baqing County: (a) The warm season and (b) The cold season. Δ indicates the coefficient of overlap, and *p* denotes the probability of temporal activity overlap between species.

While the overall activity patterns were broadly similar between the warm and cold seasons, some differences in activity peaks were observed. In the warm season, TF showed moderate temporal overlap with the PP (Δ = 0.76, CI: 0.77–0.79, *p <* 0.001) and a high degree of overlap with GP (Δ = 0.803, *p <* 0.001). In contrast, the RF showed low overlap with both the PP (Δ = 0.32, CI: 0.30–0.32, *p <* 0.001) and GP (Δ = 0.42, CI: 0.39–0.43, *p <* 0.001). In the cold season, both pika species exhibited additional minor activity peaks—PP around 15:00 and GP around 02:00, respectively. Moreover, the primary activity peak of GP shifted to approximately 13:00, occurring 1 h earlier than in the warm season. During this period, the temporal overlap between the TF and PP increased to a high level (Δ = 0.84, CI: 0.84–0.86, *p* = 0.0141), whereas the overlap between the RF and PP remained low (Δ = 0.53, CI: 0.50–0.53, *p <* 0.001). In contrast, the overlap between the TF and GP declined (Δ = 0.67, CI: 0.60–0.68, *p <* 0.001), while the overlap between the RF and GP increased to a moderate level (Δ = 0.77, CI: 0.75–0.77, *p <* 0.001) (Figure 3a,b).

### 3.3 Spatiotemporal Niche Breadth

During the warm season, TF and GP exhibited the broadest temporal niche widths (6.037 and 6.409, respectively), followed by the RF (5.731) and the PP (5.281). Conversely, during the cold season, temporal niche width decreased only for TF (4.469), whereas RF, PP, and GP all showed moderate increases (6.999, 5.642 and 6.983, respectively).

In the warm season, the RF exhibited the greatest spatial niche width (83.514), followed by the TF (21.296), GP (11.960), and PP (4.991). In the cold season, spatial niche widths declined for both fox species, reaching 50.567 for the RF and 13.904 for the TF. In contrast, GP exhibited a broader spatial niche in the cold season (14.290), whereas PP showed little seasonal variation (Table 2). Despite seasonal changes in niche width, spatial niche overlap indices (*Q*_*jk*_) remained consistently low across both seasons (*Q*_*jk*_ < 0.2) (Table S5).

**TABLE 2:** Spatiotemporal niche breadth indices of Tibetan fox, red fox, plateau pika, and Glover's pika during the cold and warm seasons in Baqing County.

		Temporal niche breadth		Spatial niche breadth	
		Warm season	Cold season	Warm season	Cold season
Tibetan fox	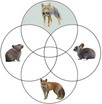	6.037	4.465	21.296	13.904
Red fox	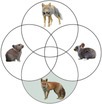	5.731	6.999	83.514	50.567
Plateau pika	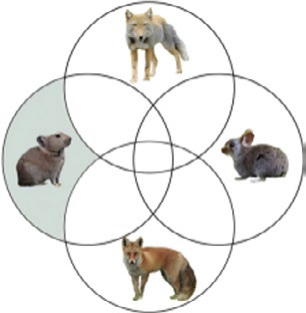	5.281	5.642	4.991	4.778
Glover's pika	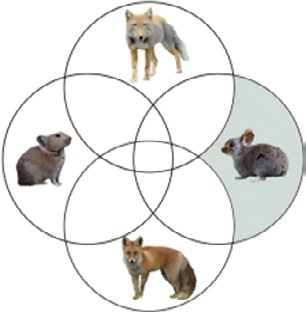	6.409	6.983	11.96	14.290

### 3.4 Marginal Occupancy Modeling

**FIGURE 4:**
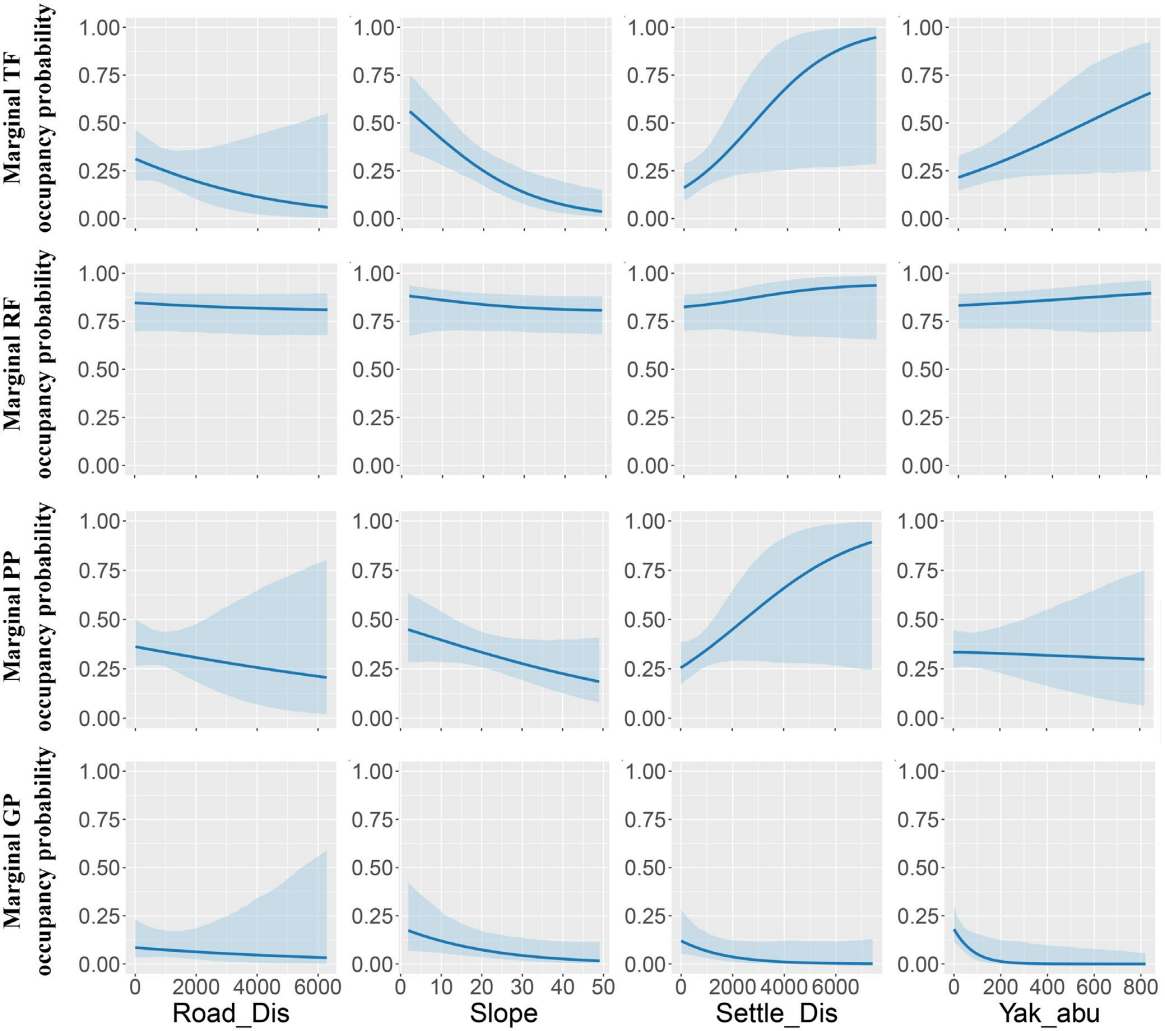
Marginal occupancy probabilities of the Tibetan fox (row 1), red fox (row 2), plateau pika (row 3), and Glover's pika (row 4) in Baqing County. Each panel shows species‐specific marginal occupancy probabilities in relation to distance to roads (column 1), slope (column 2), distance to settlements (column 3), and yak abundance (column 4). Solid lines represent posterior means, and shaded blue bands indicate 95% credible intervals.

The dataset included 170 camera trap sites, with 167 Tibetan fox, 796 red fox, 1205 plateau pika, and 885 Glover's pika detections recorded in total. The four species exhibited varying relationships between marginal occupancy probability and specific environmental factors, with significant effects observed for several species (Figure 4). For the TF, marginal occupancy probability showed a significant negative relationship with slope and distance to settlements, suggesting that increasing slope and proximity to settlements decreased the likelihood of occupancy (*p <* 0.05, Table S1). Similarly, for the RF, slope was significantly associated with decreased marginal occupancy probability, indicating a preference for flatter terrain (*p <* 0.05). Furthermore, the occupancy probability of GP in our study area was negatively related to both yak abundance and average slope, implying that higher yak densities might affect GP occupancy (*p* < 0.05). No significant relationships were observed between marginal occupancy probability and other environmental covariates for PP.

### 3.5 Conditional Occupancy Models

**FIGURE 5:**
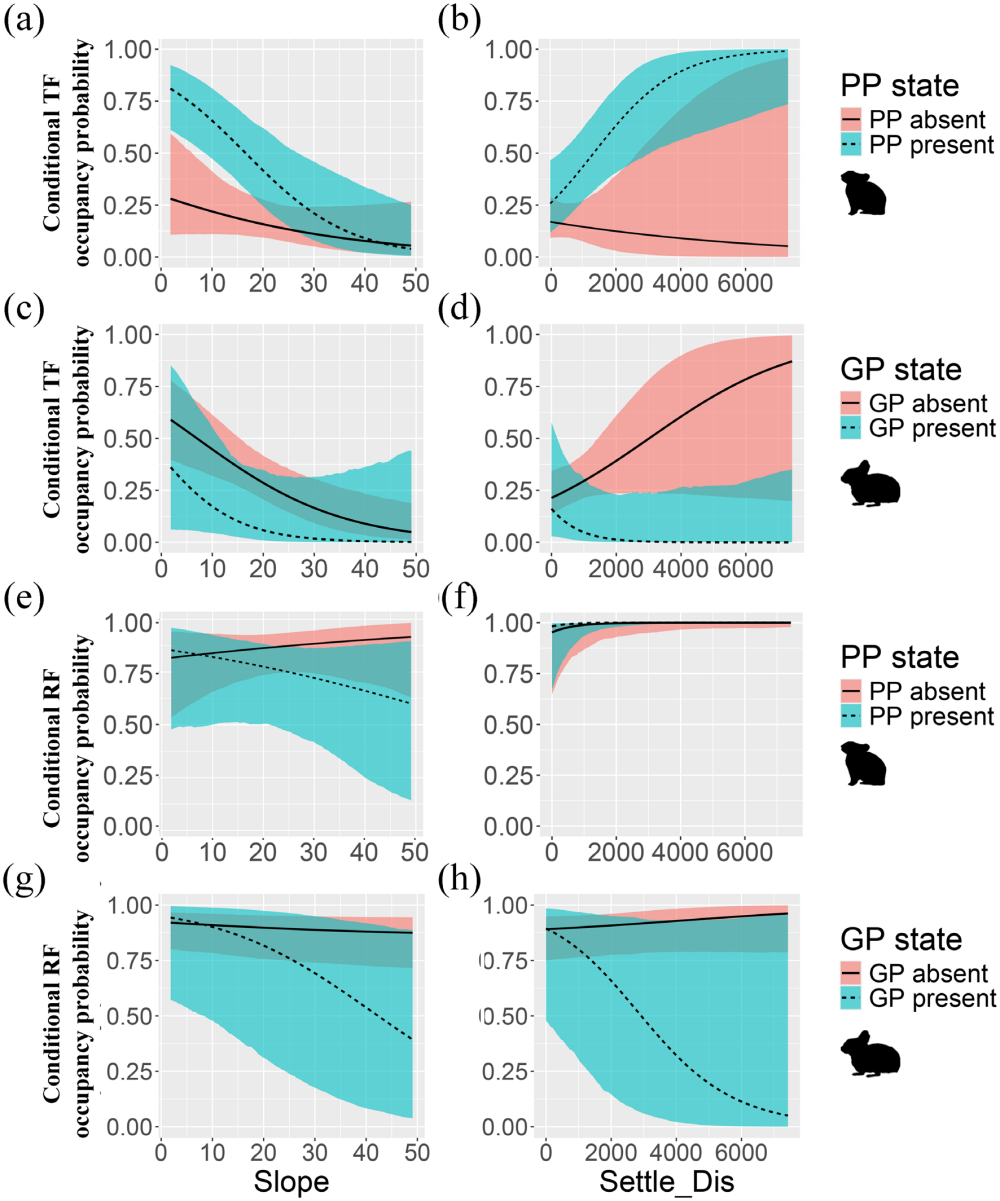
Conditional occupancy probabilities of the Tibetan fox and red fox depending on the presence (1) or absence (0) of other species. Each panel shows how the occupancy probabilities of the Tibetan fox and red fox vary with the presence (1) or absence (0) of another species. Column values reflect the occupancy probabilities of the Tibetan fox and red fox, conditioned on the species shown in the corresponding row: plateau pika (a–b and e–f), Glover's pika (c–d and g–h). Solid lines indicate posterior means, and shaded ribbons represent 95% credible intervals.

The conditional occupancy of the two fox species was further analyzed in relation to the presence or absence of their primary prey. Based on the best‐supported model, we found significant evidence for interspecific interactions among species pairs. Specifically, the conditional occupancy probability of TF was significantly influenced by the presence of PP and GP. TF occupancy was also affected by distance to human settlements, with occupancy probability increasing as the distance from settlements increased (Figure 4). Considering the presence or absence of the two pika species, TF was more likely to occupy a given area when PP was present, as distance from settlements increased and slope decreased (Figure 5a,b). In contrast, TF occupancy significantly decreased when PP was absent. These interactions were evident from the differing slopes of conditional occupancy probability lines for each species pair, with TF occupancy probability declining significantly in areas where GP was present (Figure 5d, Figure S1), indicating a stronger preference for PP as prey.

**FIGURE 6:**
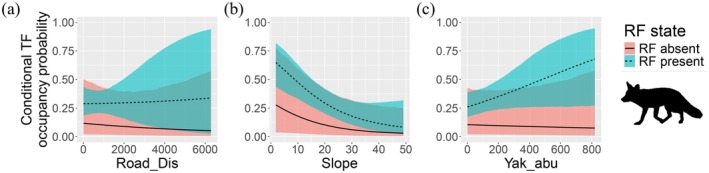
Conditional occupancy probabilities of the Tibetan fox given the presence (1) or absence (0) of the red fox in relation to Road_Dis (a), Slope (b), and Yak_abu (c). Each panel shows how the occupancy probability of the Tibetan fox varies with the presence (1) or absence (0) of the red fox. Column values reflect the occupancy probability of the Tibetan fox, conditioned on the species shown in the corresponding row: red fox. Solid lines indicate posterior means, and shaded ribbons represent 95% credible intervals.

The red fox maintained relatively high occupancy in areas farther from human settlements and on steeper slopes. However, this occupancy declined in the presence of PP and GP, indicating that the presence or absence of these two pika species had a relatively weak effect on red fox occupancy (Figure 5e,g,h). Additionally, when the presence or absence of the RF was incorporated into the model, we found that Tibetan fox occupancy was higher in areas where red foxes were present; however, this pattern was not statistically significant (Table S4). This result is consistent with our previous findings on spatial niche overlap, suggesting that spatial‐scale differentiation may represent a niche partitioning strategy between these two sympatric carnivore species (Figure 6).

## 4 Discussion

### 4.1 Seasonal Dynamics of Prey and Predators' Behavioral Responses

Prey activity intensity, food resource availability, and environmental conditions are key factors that jointly shape the spatial patterns of carnivores, activity rhythms, and coexistence (Parsons et al. 2022, 2019). Our results showed that the activity intensity of plateau pikas and Glover's pikas exhibited pronounced seasonal variation between the cold and warm seasons, which was closely associated with alpine environmental factors, including temperature, snow cover, and the availability of food resources.

In the warm season, suitable temperatures and lush vegetation provided more abundant food resources for pikas. Moreover, breeding primarily occurred from April to July. To meet the increased energetic demands of reproduction and offspring rearing, pikas exhibited more frequent foraging and higher activity levels during this period (Qu et al. 2012). By contrast, pikas adapted to winter food shortages by suppressing metabolic demands, consuming yak feces, storing hay in burrows, and avoiding predation pressure; therefore, their overall activity levels were relatively lower (Speakman et al. 2021; Morrison et al. 2009).

Thus, reduced prey activity and detectability during the cold season may alter fox space use or movement patterns (e.g., increased investment in foraging time), thereby indirectly increasing the probability of predator detection by camera‐trap records and resulting in higher detection rates in camera trap records. Similar behavioral responses have been observed in other carnivores. When the activity intensity of primary ungulate prey was low, the endangered dhole ( *Cuon alpinus* ) expanded its hunting period and increased hunting frequency to meet its energetic requirements (Havmøller, Wahyudi, et al. 2024). A similar predator–prey activity coupling was also observed between the coyote and white‐tailed deer ( *Odocoileus virginianus* ) (Kellner et al. 2022), further supporting the findings of the present study.

Although camera traps have been widely used in wildlife population monitoring, relative abundance indices derived from camera trap data may be biased by variation in detection probability, camera placement, and sensor sensitivity (Sollmann et al. 2013). To address these limitations, several new analytical approaches have been developed in recent years. Harris et al. (2024) integrated camera trap data with N‐mixture models, enabling more robust estimation of population abundance by explicitly accounting for imperfect detection. Moreover, researchers further incorporated simulations of individual movement processes and combined them with machine‐learning algorithms, such as random forests, to identify animal density patterns that best match field observations (Li et al. 2022). As research has increasingly expanded to the landscape scale, camera traps have shown considerable potential for estimating landscape‐level animal density and biomass, and for revealing relationships between the density of sympatric species and environmental factors (Nakashima et al. 2020). Collectively, the application of these emerging methods has facilitated the reduction of biases inherent in camera‐trap monitoring and reflected important implications for wildlife conservation, population management, and ecological processes studies.

### 4.2 Seasonal Modulation of Predator–Prey Temporal Coupling

In this study, diel activity rhythm analyses indicated that the red foxes were nocturnal and the Tibetan fox was diurnal, consistent with previous findings (Wang et al. 2022; Doncaster and Macdonald 1997). Carnivores often exhibited pronounced differences in their activity rhythms, and such temporal variation played an important role in facilitating species coexistence. By partitioning activity in the temporal dimension, sympatric carnivores can reduce direct encounters and interference competition (Halle 2000). In prior studies, Havmøller et al. (2020) showed that, under the competitive pressure exerted by sympatric spotted hyenas ( *Crocuta crocuta* ) and their influence on patterns of habitat resource use, male and female leopards ( *Panthera pardus* ) exhibited pronounced sex‐specific segregation in activity patterns, with males being more nocturnal than females.

Therefore, the differentiation in diel activity patterns between red foxes and Tibetan foxes may represent an adaptive strategy at a temporal scale. Although mammalian activity rhythms were primarily governed by endogenous mechanisms, they were also modulated by exogenous factors such as predation risk and resource distribution (Aschoff 1960; Weinert 2005). To increase hunting success, predators adjusted their activity rhythms to synchronize with those of their prey (de Matos Dias et al. 2018; Foster et al. 2013). In European mesocarnivore communities, most species relied on prey activity for detection; consequently, strong temporal synchrony and overlap were often observed between the activity patterns of predators and their prey (Monterroso et al. 2013).

Tibetan foxes were observed to be active during the daytime, exhibiting a high degree of temporal overlap with plateau pika (Δ = 0.961) and Glover's pika (Δ = 0.902) during the warm season, indicating a strong temporal coupling between predators and prey. In the cold season, the activity peak of Tibetan fox shifted slightly later, possibly linked to a delayed peak activity of plateau pika under low‐temperature conditions. In contrast, red foxes exhibited lower overlap with both plateau pika and Glover's pika (Δ < 0.5) during the warm season.

However, in the cold season, the decline in vegetation productivity led to a substantial reduction in the availability of alternative food resources for red foxes, such as insects, small birds, and plant‐based materials. At the same time, herders moved to winter settlements and tourist activities ceased, resulting in a marked decrease in human activity within the study area. This reduction further diminished human‐provided food sources (e.g., kitchen waste, tourist leftovers, and livestock carcasses), making scavenging more difficult for red foxes. Moreover, pikas exhibited reduced diel activity plasticity in winter, forcing red foxes to allocate more time to foraging due to energy constraints. Collectively, these factors increased the temporal overlap between red foxes and pikas. Consequently, temporal overlap between red fox and Glover's pika was higher in the cold season than in the warm season, suggesting that red foxes adjust their foraging behavior in response to seasonal changes in food availability. Overall, the temporal niche overlap between the Tibetan fox and the red fox shifted dynamically across seasons, reflecting both the complexity of predator–prey interactions and the adaptive flexibility of predators in response to environmental changes in the plateau ecosystem.

### 4.3 Prey‐Mediated Spatial Association and Avoidance in Carnivores

Spatial associations between predators and their prey are often shaped by prey preferences, leading predators to exhibit distinct spatial patterns in response to different prey species (Gorini et al. 2011). As shown by the occupancy model results, Tibetan foxes exhibited a highly specialized dependence on plateau pika, making them highly sensitive to changes in pika populations and habitat conditions (Zhang et al. 2025). Consequently, their occupancy probability was higher in areas where plateau pika are present, reflecting the importance of plateau pika as a primary prey species (Zheng et al. 2023). Habitats characterized by gentle slopes and sparse, short‐statured vegetation tend to support higher pika abundance, and the openness of these landscapes likely enhances Tibetan fox hunting efficiency (Smith and Foggin 1999).

Interestingly, our results revealed a geographic pattern of spatial and interspecific dependence between Tibetan fox and red fox. In Baqing County, after accounting for environmental variables, the distributions of Tibetan fox and red fox were positively correlated. Similar co‐occurrence patterns have been reported by Rota et al. (2016), where red fox occupancy at camera sites was higher when coyotes were present, as observed in northern Virginia and Maryland. It's speculated that the prey‐mediated coexistence may be a mechanism for positive spatial associations between two canid species (Lesmeister et al. 2015). This also supports previous findings that areas where both fox species coexist likely had relatively high pika abundance, suggesting that the two species can share foraging habitats without mutual exclusion (Tsukada et al. 2014).

Moreover, anthropogenic disturbances were key factors influencing the spatial distribution patterns of the two canid species, particularly the distribution of roads and settlements. Previous studies reported that Tibetan fox exhibited a marked avoidance of human disturbance, with significantly higher activity levels in areas farther from roads and settlements (Gong and Hu 2003). Although our results indicated that Tibetan fox was more likely to occupy areas closer to roads, it still occurred at quite low occupancy. Compared with the Tibetan fox, the red fox appeared highly adapted to human environments, showing less influence from slopes, roads and settlements, while maintaining a relatively high occupancy rate. This finding was consistent with Barros et al. (2024) in that the red fox, as a generalist species, seemed able to shift in response to environmental stressors. This distribution pattern may be linked to the red fox's broader diet and dietary flexibility. Prior studies have indicated that red foxes may obtain food subsidies from human activities along roads, such as edible waste discarded by drivers and the carcasses of road‐killed animals (Forman and Alexander 1998; Balestrieri et al. 2011). Thus, the red fox may alleviate interspecific resource competition with the Tibetan fox by foraging in areas with greater human influence. Similar phenomena occurred in brown bears comparing with hunting natural prey (i.e., pikas and Himalayan marmots); brown bears also showed a greater tendency to exploit human‐derived food resources that provided higher energy and lower foraging costs (Li et al. 2025; Dai et al. 2021). According to optimal foraging theory, predators maximize their net energy intake by selecting food resources and foraging habitats that offered the highest energetic returns while minimizing acquisition costs and predation risk (Pyke 1984). Therefore, wildlife behavioral patterns may be influenced by anthropogenic disturbance, making this an issue that warrants continued attention.

The occupancy of two fox species appeared to be marginally affected by the presence or absence of Glover's pika. This pattern is consistent with differences in spatial use and dietary niches between the two fox species. Prior studies indicated significant differences in prey use between the two fox species, that is, plateau pika DNA was detected in 134 of 135 fecal samples from Tibetan fox, and 98 samples (73%) contained only pika remains (Harris et al. 2014). The Tibetan fox exhibited a relatively specialized foraging strategy, relying heavily on plateau pika. In contrast, the red fox had a broader diet (Shrotriya et al. 2022). Nevertheless, Glover's pika constituted only a small proportion of the diet of both fox species (Shrotriya et al. 2022; Harris et al. 2014). This may be closely related to the behavioral ecology of Glover's pika, as the species typically inhabits structurally complex microhabitats such as rock crevices and talus slopes (Jiang et al. 2017), which greatly enhances its ability to evade predation and increases the hunting costs and failure rates for predators such as foxes.

Furthermore, pikas are preyed upon by nearly all of the predators occurring on the Tibetan Plateau. These predators include wolves ( *Canis lupus* ), red foxes, Tibetan foxes, snow leopards ( *Panthera uncia* ), and raptors (Wei et al. 2020). Previous studies have demonstrated that pikas are a main food source of the Saker falcon ( *Falco cherrug* ) (90% of pellets collected beneath Saker falcon nests contained pika remains) (Dixon et al. 2015). The reliance of multiple predators on a shared prey resource may enhance interspecific competition. According to optimal foraging and resource competition theory, predators balance foraging efficiency and predation risk while maximizing energetic returns, thereby reducing spatial overlap and direct conflicts with other predators and adopting a spatial “avoidance” strategy in their foraging behavior (Pyke 1984; Pyke et al. 1977). Foxes and wolves were indicated to exhibit substantial spatial overlap but clear temporal segregation in their activity patterns, and fox activity intensity was negatively correlated with wolf pack size. In addition, wolves can directly kill foxes, which forced them to avoid areas with high wolf activity (Wikenros et al. 2017). Therefore, under competitive pressure from multiple predators, we inferred that red foxes and Tibetan foxes likely balanced foraging efficiency by actively avoiding areas used by other predators to reduce predation risk, which in turn influenced the allocation of prey resources (Ritchie and Johnson 2009). Overall, the relatively low use of Glover's pika by foxes may be related not only to the concealed microhabitats of pikas but also to interspecific interactions among predators.

Although our spatiotemporal co‐occurrence analysis provided novel insights into overlap patterns between the two carnivore species, it did not yet allow for joint inference of spatial and temporal interactions. Recently, Havmøller, Parsons, et al. (2024) applied a multispecies occupancy model with a continuous‐time detection process, revealing the daily activity patterns of male and female leopards in the presence or absence of hyenas. Further studies should incorporate both temporal and spatial interactions into the analytical models to achieve a more comprehensive understanding of species coexistence mechanisms.

### 4.4 Functional Stability of the Plateau Food Web and Conservation Strategies

Both Tibetan fox and red fox played vital ecological roles in the QTP ecosystem, yet they exhibited distinct interspecific differences in their responses to prey resource availability and anthropogenic disturbance. The Tibetan fox, an endemic species of the QTP, fulfilled an irreplaceable functional role in regulating plateau pika populations and mitigating the impacts of regional “pika outbreaks” (Schaller 2000). However, contemporary anthropogenic pressures, including large‐scale poisoning campaigns targeting plateau pikas (a common control strategy), grassland degradation driven by overgrazing, and habitat fragmentation associated with road and infrastructure expansion, were systematically eroding the primary prey resources and core foraging habitats on which Tibetan foxes depend for survival (Harris et al. 2014). Furthermore, our findings revealed that Tibetan fox exhibited relatively low relative abundance indices (RAI) and heightened sensitivity to human disturbance. In addition, long‐term high parasitic loads may further compromise their physiological fitness, rendering them more vulnerable to adverse environmental conditions under resource‐limited scenarios (Chen et al. 2025). In contrast, the red fox, characterized by a broader dietary niche and higher disturbance tolerance, demonstrated greater adaptive capacity to habitat modification and mild fragmentation.

Given these interspecific differences in dietary specialization, parasitic vulnerability, and disturbance resilience, conservation and management strategies should be species‐specific to ensure their effectiveness. For the diet‐specialized Tibetan fox, priority measures should include: (1) enhancing long‐term monitoring and early warning systems for both Tibetan fox populations and their primary prey (plateau pikas); (2) protecting and restoring critical suitable habitats; (3) mitigating habitat fragmentation; and (4) promoting sustainable grassland management practices. These integrated measures would be essential for maintaining stable Tibetan fox populations. Conversely, the red fox's high tolerance to human disturbance and propensity for human‐wildlife interactions necessitate targeted management of anthropogenic subsidies (Figure S2). In particular, unauthorized feeding associated with tourism can alter red fox foraging behavior, disrupt natural foraging abilities, induce dependence on artificial food, and ultimately diminish their ecological function in regulating lagomorph and rodent populations. Strict prohibition of unauthorized feeding is necessary to mitigate risks associated with human‐wildlife interactions, thereby safeguarding positive trophic interactions within the QTP wildlife food web and sustaining the functional stability of this high‐altitude ecosystem.

## 5 Conclusion

Spatiotemporal niche partitioning was evident among Tibetan foxes, red foxes, plateau pikas, and Glover's pikas. The two fox species showed contrasting diel activity patterns and diverged in their responses to key environmental variables. Tibetan foxes displayed a strong ecological association with plateau pikas and exhibited pronounced avoidance of human‐disturbed habitats. In contrast, red foxes showed greater habitat generalism but avoided prey‐rich patches where both pika species co‐occurred. These interspecific differences likely reflect species‐specific ecological strategies shaped by prey availability and disturbance tolerance, underscoring the critical role of spatiotemporal differentiation, prey selectivity, and anthropogenic disturbance in facilitating sympatric coexistence. Our findings advance the understanding of predator–prey interactions in high‐elevation ecosystems and provide new insights for biodiversity conservation and adaptive management on the QTP.

## Author Contributions

**Yuanzhen Cui:** data curation (equal), formal analysis (equal), investigation (equal), methodology (equal), writing – original draft (equal). **Dandan Wang:** data curation (equal), formal analysis (equal), methodology (equal), writing – original draft (equal). **Fuxing Huang:** data curation (equal), methodology (equal). **Zhiming Cao:** data curation (equal). **Yuqin Liu:** data curation (equal), methodology (equal). **Awang Pingcuo:** data curation (equal), methodology (equal). **Da Qiong:** data curation (equal), methodology (equal). **Xiaolong Hu:** methodology (equal). **Yongtao Xu:** formal analysis (lead), investigation (lead), methodology (lead), resources (lead), supervision (lead), validation (lead).

## Funding

This work was supported by the National Natural Science Foundation of China (No. 32470552), the Natural Science Foundation of Jiangxi Province, China (No. 20242BAB25349), and the Implementation Plan Procurement Project of the Forestry and Grassland Ecological Protection and Restoration Fund (First Batch of National Park Subsidy) of Naqu in 2022 (No. GZFCG2023‐15109).

## Conflicts of Interest

The authors declare no conflicts of interest.

## Supporting information


**Table S1:** Marginal occupancy probability estimates of four species predicted from a multi‐species occupancy model, including coefficients (Estimate), standard errors (SE), z‐values (z), and corresponding *p*‐values (P(>|z|)) for each species: TF = Tibetan Fox, RF = Red Fox, PP = Plateau Pika, GP = Glover's Pika. Covariates include slope (Slope), distance to settlements (Settle_Dis), distance to roads (Road_Dis), and yak abundance (Yak_abu). Significant effects (*p* < 0.05) are highlighted in bold.
**Table S2:** Estimated intercepts (on the logit scale) of marginal occupancy probabilities and pairwise co‐occurrence states derived from the multi‐species occupancy model.
**Table S3:** Model selection was conducted using a multi‐species occupancy model. Candidate models were ranked by AIC, with the top model highlighted in bold. For each species pair (TF‐RF, TF‐PP, TF‐GP, RF‐PP, RF‐GP), we used all six covariates (i.e., Road_Dis; Settle_Dis; Aspect; Slope; Altitude; Yak_abu) for natural parameters—distance to the nearest road, distance to the nearest settlement, aspect, slope, altitude, and yak relative abundance. All co‐occurrence models for each species pair were fitted, and the 10 highest‐ranking models were retained for comparison. Models denoted ~1 are intercept‐only models.
**Table S4:** Estimated effects of environmental covariates on pairwise species co‐occurrence probabilities (ψ) from the multi‐species occupancy model. Each row shows the estimate (Estimate), standard error (SE), and 95% confidence interval (Lower Confidence Limit, LCL; Upper Confidence Limit, UCL) for a given species pair and covariates. Estimates with confidence intervals that do not overlap zero indicate statistically meaningful effects on co‐occurrence probabilities.
**Table S5:** Spatial niche overlap of Tibetan fox, red fox, plateau pika, and glover's pika during the cold and warm seasons in Baqing County.
**Figure S1:** Study area of mino‐carnivores and main preys: (a) The boundary of Baqing County (b) The ecological photo of warm season (c) The habitat in cold season.
**Figure S2:** Red fox actively approaching passing vehicles and accepting food provisioned by humans (S2a, S2b, S2c), indicating food‐seeking behavior associated with human activities (S2d).

## Data Availability

The raw data and scripts used in this study are available from Dryad at https://doi.org/10.5061/dryad.q2bvq840n. Scripts can also be found in Rota et al. (2016) at https://doi.org/10.1111/2041‐210X.12587.
