# Supplementary material for: Spatiotemporal Distribution Patterns of Plateau Foxes and Their Lagomorph Prey in Baqing County, Tibet
Source: Ecol Evol. 2026 Mar 30;16(4):e73361. doi: 10.1002/ece3.73361 (PMC13107289; doi:10.1002/ece3.73361)

**Supplementary Table S1:** Marginal occupancy probability estimates of four species predicted from a multi‐species occupancy model, including coefficients (Estimate), standard errors (SE), z-values (z), and corresponding p-values (P(>|z|)) for each species: TF = Tibetan Fox, RF = Red Fox, PP = Plateau Pika, GP = Glover’s Pika. Covariates include slope (Slope), distance to settlements (Settle_Dis), distance to roads (Road_Dis), and yak abundance (Yak_abu). Significant effects (*p* < 0.05) are highlighted in bold.

|  | **Estimate** | **SE** | **z** | **P(>\|z\|)** |
| --- | --- | --- | --- | --- |
| [TF] (Intercept) | -2.448436 | 1.300147 | -1.8832 | 5.97E-02 |
| **[TF] Slope** | **-0.074188** | **0.021884** | **-3.39** | **6.99E-04** |
| **[TF] Settle_Dis** | **0.000612** | **0.000293** | **2.0896** | **3.67E-02** |
| [TF] Road_Dis | -0.000316 | 0.000281 | -1.1256 | 2.60E-01 |
| [TF] Yak_abu | 0.002371 | 0.001298 | 1.8263 | 6.78E-02 |
| [RF] (Intercept) | -1.528526 | 1.164851 | -1.312 | 1.89E-01 |
| **[RF] Slope** | **0.151009** | **0.074308** | **2.032** | **4.21E-02** |
| [RF] Settle_Dis | 0.000276 | 0.000737 | 0.375 | 7.08E-01 |
| [RF] Road_Dis | 0.001052 | 0.000568 | 1.852 | 6.40E-02 |
| [RF] Yak_abu | 0.469508 | 0.414103 | 1.134 | 2.57E-01 |
| [PP] (Intercept) | -0.224393 | 0.703059 | -0.319 | 7.50E-01 |
| [PP] Slope | -0.027456 | 0.01732 | -1.585 | 1.13E-01 |
| [PP] Settle_Dis | 0.000428 | 0.000256 | 1.674 | 9.42E-02 |
| [PP] Road_Dis | -0.000126 | 0.00024 | -0.524 | 6.00E-01 |
| [PP] Yak_abu | -0.000229 | 0.001295 | -0.177 | 8.59E-01 |
| [GP] (Intercept) | 1.118172 | 0.790547 | 1.414 | 1.57E-01 |
| **[GP] Slope** | **-0.054598** | **0.026741** | **-2.042** | **4.12E-02** |
| [GP] Settle_Dis | -0.000626 | 0.000371 | -1.686 | 9.18E-02 |
| [GP] Road_Dis | -0.000163 | 0.000342 | -0.478 | 6.33E-01 |
| **[GP] Yak_abu** | **-0.014002** | **0.00656** | **-2.135** | **3.28E-02** |

**Supplementary Table S2:** Estimated intercepts (on the logit scale) of marginal occupancy probabilities and pairwise co-occurrence states derived from the multi-species occupancy model.

|  | **Estimate** | **SE** | **z** | **P(>\|z\|)** |
| --- | --- | --- | --- | --- |
| [TF] (Intercept) | **-3.4136** | 1.264 | -2.7013 | **6.91E-03** |
| [RF] (Intercept) | **1.9228** | 0.436 | 4.4057 | **1.05E-05** |
| [PP] (Intercept) | -0.3561 | 0.541 | -0.6589 | 5.10E-01 |
| [GP] (Intercept) | -0.8301 | 0.61 | -1.3619 | 1.73E-01 |
| [TF:RF] (Intercept) | 1.9695 | 1.248 | 1.5786 | 1.14E-01 |
| [TF:PP] (Intercept) | **2.1255** | 0.495 | 4.2969 | **1.73E-05** |
| [TF:GP] (Intercept) | -0.8712 | 0.754 | -1.1552 | 2.48E-01 |
| [RF:PP] (Intercept) | -1.2572 | 0.649 | -1.9375 | 5.27E-02 |
| [RF:GP] (Intercept) | -0.8412 | 0.709 | -1.1872 | 2.35E-01 |
| [PP:GP] (Intercept) | -0.0367 | 0.572 | -0.0642 | 9.49E-01 |

**Supplementary Table S3:** Model selection was conducted using a multi-species occupancy model. Candidate models were ranked by AIC, with the top model highlighted in bold. For each species pair (TF-RF, TF-PP, TF-GP, RF-PP, RF-GP), we used all six covariates six covariates (i.e., Road_Dis; Settle_Dis; Aspect; Slope; Altitude; Yak_abu) for natural parameters—distance to the nearest road, distance to the nearest settlement, aspect, slope, altitude, and yak relative abundance. All co-occurrence models for each species pair were fitted, and the ten highest-ranking models were retained for comparison. Models denoted ~1 are intercept-only models.

| **Species** | **Model description** | **AIC** | **Delta AIC** | **Species pair** |
| --- | --- | --- | --- | --- |
| **TF** | **~Road_Dis+Slope+Yak_abu** | **2369.534** | **0** | **RF** |
| TF | ~Road_Dis+Slope | 2369.923 | 0.389 | RF |
| TF | ~Yak_abu | 2377.835 | 8.301 | RF |
| TF | ~1 | 2378.453 | 8.919 | RF |
| TF | ~Aspect+Yak_abu | 2379.818 | 10.284 | RF |
| TF | ~Aspect | 2380.443 | 10.909 | RF |
| TF | ~Road_Dis+Yak_abu | 2381.158 | 11.624 | RF |
| TF | ~Road_Dis | 2381.172 | 11.638 | RF |
| TF | ~Road_Dis+Aspect+Yak_abu | 2383.332 | 13.798 | RF |
| TF | ~Settle_Dis | 2679.084 | 309.55 | RF |
| **TF** | **~Road_Dis+Settle_Dis+Slope** | **2363.634** | **0** | **PP** |
| TF | ~Road_Dis+Settle_Dis+Slope+Yak_abu | 2363.708 | 0.074 | PP |
| TF | ~Road_Dis+Settle_Dis+Aspect+Slope | 2365.307 | 1.673 | PP |
| TF | ~Slope+Yak_abu | 2366.24 | 2.606 | PP |
| TF | ~Aspect+Slope | 2367.234 | 3.6 | PP |
| TF | ~Aspect+Slope+Yak_abu | 2368.242 | 4.608 | PP |
| TF | ~Road_Dis+Slope | 2369.86 | 6.226 | PP |
| TF | ~Road_Dis+Slope+Yak_abu | 2370.528 | 6.894 | PP |
| TF | ~Road_Dis+Aspect+Slope | 2371.939 | 8.305 | PP |
| TF | ~Road_Dis+Settle_Dis | 2377.911 | 14.277 | PP |
| **TF** | **~Settle_Dis+Slope** | **2361.575** | **0** | **GP** |
| TF | ~Settle_Dis+Aspect | 2377.823 | 16.248 | GP |
| TF | ~Road_Dis+Settle_Dis+Aspect | 2378.399 | 16.824 | GP |
| TF | ~Settle_Dis+Yak_abu | 2378.569 | 16.994 | GP |
| TF | ~Settle_Dis+Slope+Yak_abu | 2378.652 | 17.077 | GP |
| TF | ~Settle_Dis+Aspect+Yak_abu | 2379.067 | 17.492 | GP |
| TF | ~Settle_Dis+Aspect+Slope+Yak_abu | 2379.208 | 17.633 | GP |
| TF | ~Aspect+Slope | 2379.349 | 17.774 | GP |
| TF | ~Road_Dis+Settle_Dis+Aspect+Yak_abu | 2379.46 | 17.885 | GP |
| TF | ~Slope+Yak_abu | 2379.542 | 17.967 | GP |
| **RF** | **~Settle_Dis+Slope** | **2373.068** | **0** | **PP** |
| RF | ~Settle_Dis+Aspect+Slope | 2374.976 | 1.908 | PP |
| RF | ~Settle_Dis+Slope+Yak_abu | 2375.434 | 2.366 | PP |
| RF | ~Settle_Dis+Aspect+Slope+Yak_abu | 2376.912 | 3.844 | PP |
| RF | ~Settle_Dis+Aspect | 2378.781 | 5.713 | PP |
| RF | ~Settle_Dis+Yak_abu | 2378.91 | 5.842 | PP |
| RF | ~Settle_Dis+Aspect+Yak_abu | 2380.859 | 7.791 | PP |
| RF | ~Aspect+Yak_abu | 2382.41 | 9.342 | PP |
| RF | ~Road_Dis+Settle_Dis | 2681.084 | 308.016 | PP |
| RF | ~Road_Dis+Aspect | 2681.084 | 308.016 | PP |
| **RF** | **~Settle_Dis+Slope+Yak_abu** | **2374.547** | **0** | **GP** |
| RF | ~Settle_Dis+Aspect+Slope+Yak_abu | 2375.756 | 1.209 | GP |
| RF | ~Aspect+Yak_abu | 2376.949 | 2.402 | GP |
| RF | ~Settle_Dis+Yak_abu | 2378.154 | 3.607 | GP |
| RF | ~Road_Dis+Settle_Dis+Slope+Yak_abu | 2378.434 | 3.887 | GP |
| RF | ~Road_Dis+Slope+Yak_abu | 2378.598 | 4.051 | GP |
| RF | ~Road_Dis+Slope+Aspect+Yak_abu | 2379.03 | 4.483 | GP |
| RF | ~Settle_Dis+Aspect+Yak_abu | 2379.294 | 4.747 | GP |
| RF | ~Road_Dis+Aspect+Yak_abu | 2380.317 | 5.77 | GP |
| RF | ~Road_Dis+Settle_Dis+Yak_abu | 2380.448 | 5.901 | GP |

**Supplementary Table S4:** Estimated effects of environmental covariates on pairwise species co-occurrence probabilities (ψ) from the multi-species occupancy model. Each row shows the estimate (Estimate), standard error (SE), and 95% confidence interval (Lower Confidence Limit, LCL; Upper Confidence Limit, UCL) for a given species pair and covariates. Estimates with confidence intervals that do not overlap zero indicate statistically meaningful effects on co-occurrence probabilities.

| **Parameter** | **Estimate** | **SE** | **LCL**  **（Lower Confidence Limit）** | **UCL**  **（Upper Confidence Limit）** |
| --- | --- | --- | --- | --- |
| psi([TF:RF] Road_Dis) | 0.000097 | 0.000307 | −0.000504 | 0.000698 |
| psi([TF:RF] Slope) | −0.028200 | 0.027632 | −0.082341 | 0.025976 |
| psi([TF:RF] Yak_abu) | 0.002600 | 0.001599 | −0.000537 | 0.005731 |
| psi([TF:PP] Road_Dis) | −0.000554 | 0.000370 | −0.001280 | 0.000172 |
| psi([TF:PP] Slope) | −0.057400 | 0.039954 | −0.135709 | 0.020896 |
| **psi([TF:PP] Settle_Dis)** | **0.000958** | **0.000472** | **0.000033** | **0.001883** |
| **psi([TF:GP] Settle_Dis)** | **−0.001769** | **0.000751** | **−0.003241** | **−0.000297** |
| psi([TF:GP] Slope) | −0.051681 | 0.079364 | −0.207236 | 0.103873 |
| psi([RF:PP] Settle_Dis) | 0.000426 | 0.000245 | −0.000054 | 0.000906 |
| **psi([RF:PP] Slope)** | **−0.047332** | **0.020602** | **−0.087711** | **−0.006953** |
| **psi([RF:GP] Settle_Dis)** | **−0.000867** | **0.000350** | **−0.001554** | **−0.000180** |
| **psi([RF:GP] Slope)** | **−0.058508** | **0.026830** | **−0.111094** | **−0.005922** |
| **psi([RF:GP] Yak_abu)** | **−0.016150** | **0.007682** | **−0.031207** | **−0.001094** |

**Supplementary Table S5:** Spatial niche overlap of Tibetan fox, red fox, plateau pika, and glover’s pika during the cold and warm seasons in Baqing County.

| ****Species**** | | **Spatial niche overlap index** | |
| --- | --- | --- | --- |
|  |  | **Warm season** | **Cold season** |
| **Tibetan fox vs Red fox** | **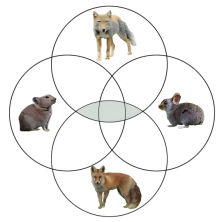** | **0.133** | **0.127** |
| **Tibetan fox vs Plateau pika** | **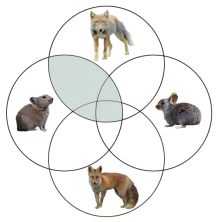** | **0.174** | **0.188** |
| **Tibetan fox vs Glover’s pika** | **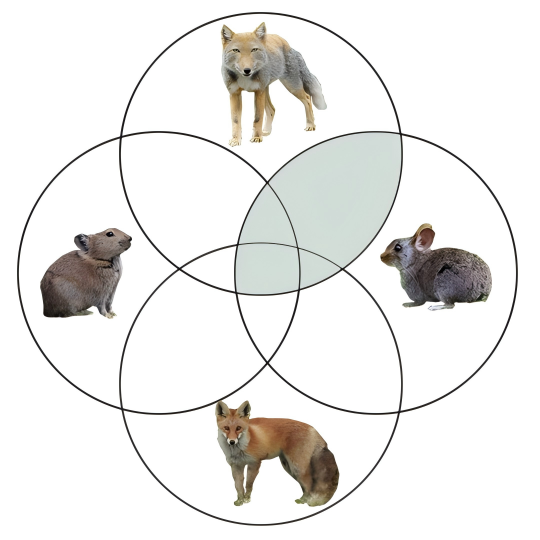** | **0.015** | **0.003** |
| **Red fox vs Plateau pika** | **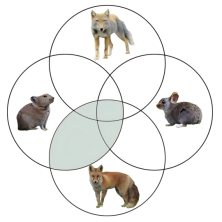** | **0.051** | **0.077** |
| **Red fox vs Glover’s pika** | **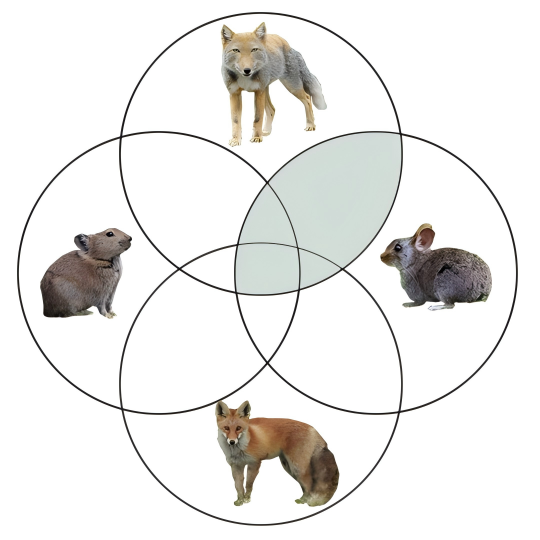** | **0.028** | **0.090** |

**Figure S1:** Study area of mino-carnivores and main preys: (a) The boundary of Baqing County (b) The ecological photo of warm season (c) The habitat in cold season.


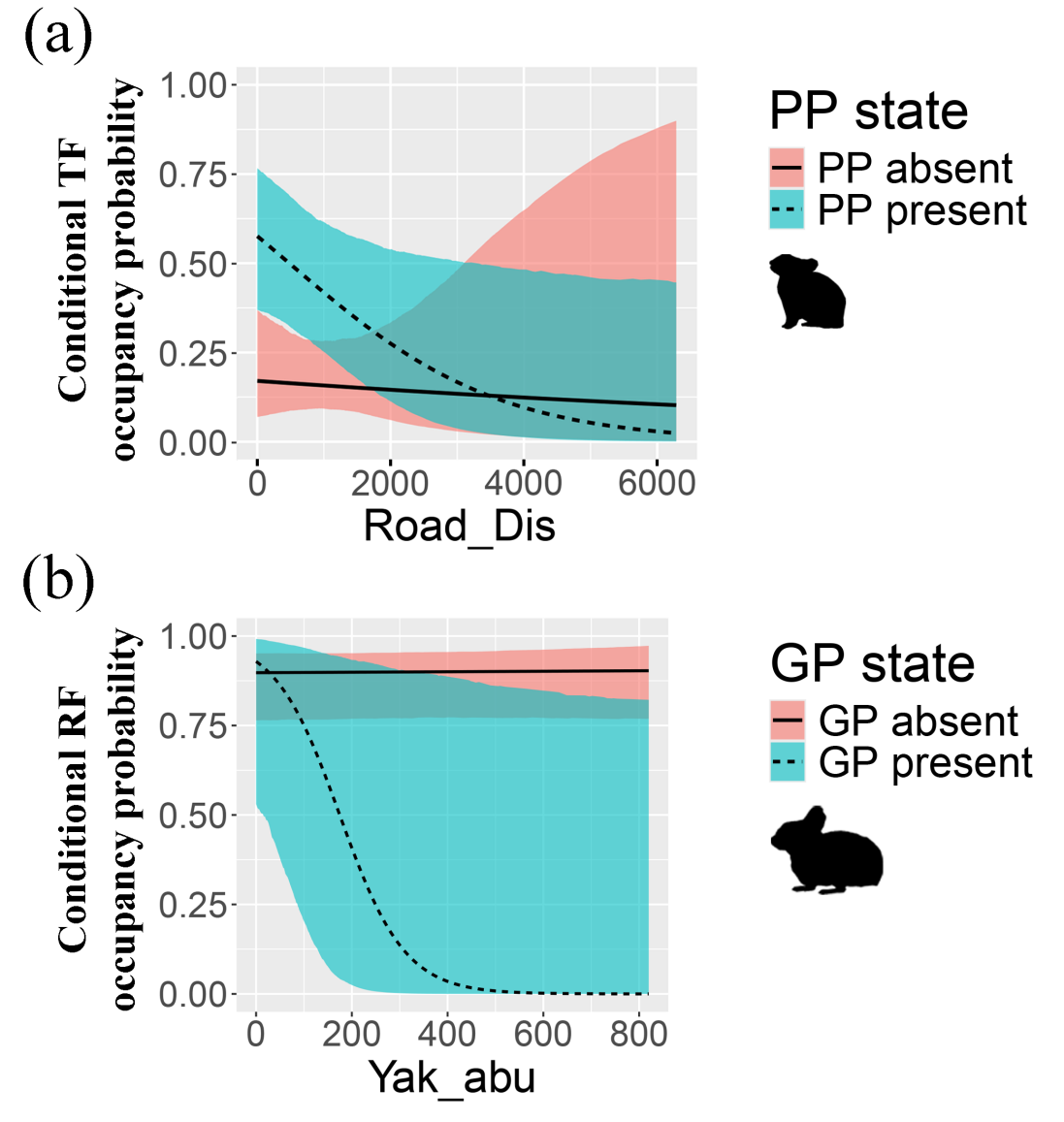


**Figure S2:** Red fox actively approaching passing vehicles and accepting food provisioned by humans (S2a, S2b, S2c), indicating food-seeking behavior associated with human activities (S2d).


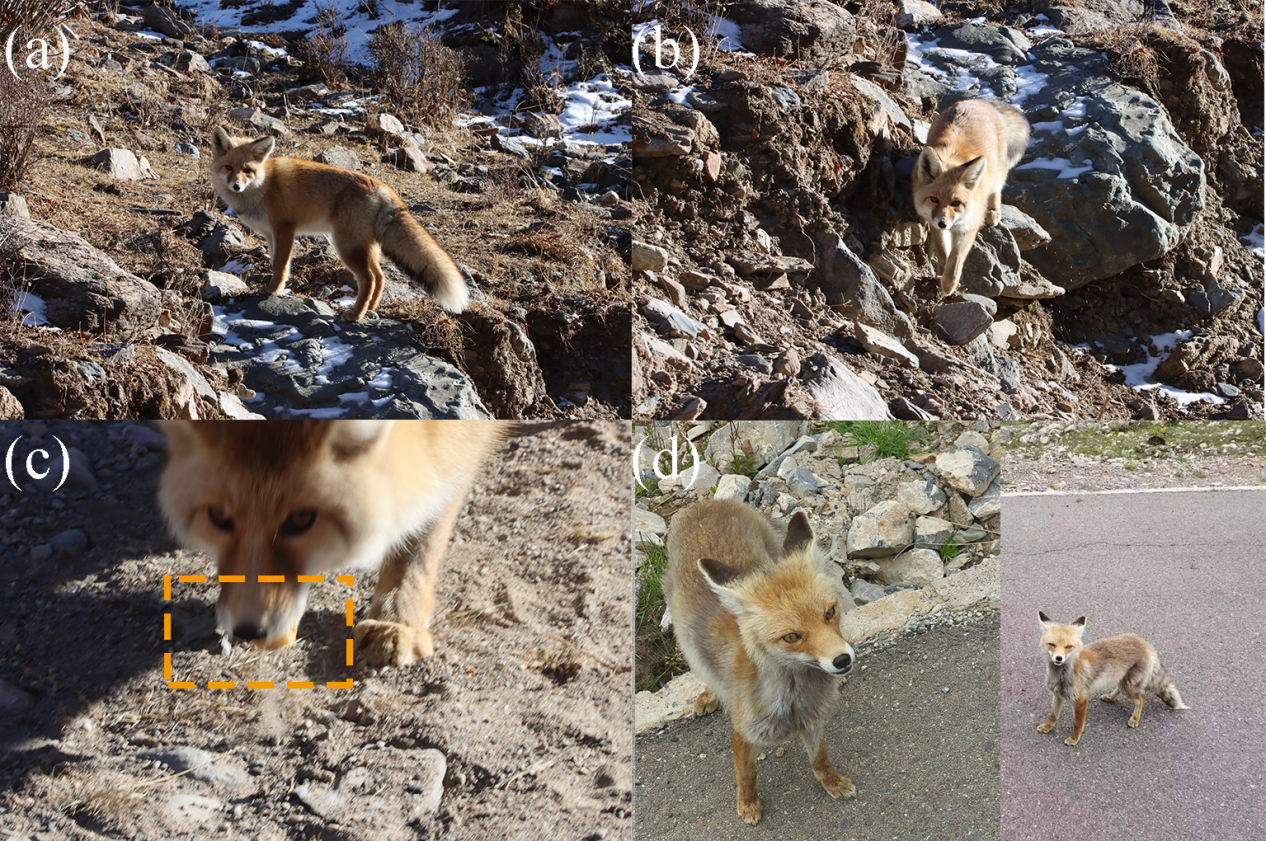

Supplement: Supplementary file 1 — Table S1: Marginal occupancy probability estimates of four species predicted from a multi‐species occupancy model, including coefficients (Estimate), standard errors (SE), z‐values (z), and corresponding p‐values (P(>|z|)) for each species: TF = Tibetan Fox, RF = Red Fox, PP = Plateau Pika, GP = Glover's Pika. Covariates include slope (Slope), distance to settlements (Settle_Dis), distance to roads (Road_Dis), and yak abundance (Yak_abu). Significant effects (p < 0.05) are highlighted in bold. Table S2: Estimated intercepts (on the logit scale) of marginal occupancy probabilities and pairwise co‐occurrence states derived from the multi‐species occupancy model. Table S3: Model selection was conducted using a multi‐species occupancy model. Candidate models were ranked by AIC, with the top model highlighted in bold. For each species pair (TF‐RF, TF‐PP, TF‐GP, RF‐PP, RF‐GP), we used all six covariates (i.e., Road_Dis; Settle_Dis; Aspect; Slope; Altitude; Yak_abu) for natural parameters—distance to the nearest road, distance to the nearest settlement, aspect, slope, altitude, and yak relative abundance. All co‐occurrence models for each species pair were fitted, and the 10 highest‐ranking models were retained for comparison. Models denoted ~1 are intercept‐only models. Table S4: Estimated effects of environmental covariates on pairwise species co‐occurrence probabilities (ψ) from the multi‐species occupancy model. Each row shows the estimate (Estimate), standard error (SE), and 95% confidence interval (Lower Confidence Limit, LCL; Upper Confidence Limit, UCL) for a given species pair and covariates. Estimates with confidence intervals that do not overlap zero indicate statistically meaningful effects on co‐occurrence probabilities. Table S5: Spatial niche overlap of Tibetan fox, red fox, plateau pika, and glover's pika during the cold and warm seasons in Baqing County. Figure S1: Study area of mino‐carnivores and main preys: (a) The boundary of Baqing County (b) The ecol [file ECE3-16-e73361-s001.docx]
